# Quantification of Bioactive Compounds and Evaluation of the Antioxidant Activity of* Carissa edulis* Valh (Apocynaceae) Leaves

**DOI:** 10.1155/2019/7549620

**Published:** 2019-05-15

**Authors:** Sabine Adeline Fanta Yadang, Germain Taiwe Sotoing, Katoucha Sidoine Ngatcha Zouakeu, Muhammad Ahsan Khan, Gabriel Agbor Agbor, Nisar Ur-Rahman, Elisabeth Ngo Bum

**Affiliations:** ^1^Institute of Medical Research and Medicinal Plants Studies, Ministry of Scientific Research and Innovation, Yaoundé, Cameroon; ^2^Department of Biological Sciences, Faculty of Science, University of Ngaoundere, Cameroon; ^3^Department of Zoology and Animal Physiology, Faculty of Science, University of Buea, Cameroon; ^4^Department of Pharmacy, Comsats University Islamabad, Abbottabad Campus, Pakistan; ^5^Institute of Mining and Petroleum Industries, University of Maroua, Cameroon

## Abstract

*Carissa edulis *is a plant used in the management of oxidative stress and inflammatory related disorders such as malaria, rheumatism inflammation, and cardiovascular diseases. The present study evaluates the total phenolic content, antioxidant capacity (DPPH, ABTS, and FRAP), and the bioactive compounds present in the various extracts of* C. edulis* (HEC, MEC, AEC, and PC). An HPLC analysis determined the different compounds present in the extracts. High concentration of total phenolic content was observed in aqueous and methanolic extracts more than in the hydroethanolic extract though not significantly different. Flavonoids were higher in the hydroethanolic and methanolic extracts, respectively, with 14.84 mg RE/g extract and 12.02 mg RE/g extract. Tannins were also found in large amounts in the same two extracts with 26.76 mg TAE/g extract and 34.67 mg TEE/g extract. The percentage radical scavenging activity DPPH ranged between 58.63% and 94.67% for aqueous extract and for ABTS between 51.39% and 94.12% for the methanolic extract. The highest FRAP was obtained in the methanolic extract (6.73 g AAE/100 g extract). HPLC analysis revealed the presence of quercetin, rutin, and gallic acid in the different extracts.* C. edulis* represents a potential source of bioactive components with antioxidant capacity.

## 1. Introduction

Mitochondrial oxidative phosphorylation, which is a process that leads to energy production, is a phenomenon that occurs in most aerobic organisms. The partial electron transfer of oxygen molecules during this metabolism leads to the production of unstable molecules such as the superoxide anion O^2-^ which is the radical form among reactive oxygen species (ROS). The ROS includes free radicals such as O^2-^, hydroxyl radical HO^−^, and also hydrogen peroxide H_2_O_2_ which is a nonradical species but is capable of liberating hydroxyl radicals according to the Fenton reaction. The increased level of these free radicals causes oxidative stress. These ROS play an important role in the physiological and pathological process. Under normal conditions, the level of free radicals is controlled by an antioxidant defense system that decreases oxidative damage [1]. Oxidative stress is considered a major contributor to many chronic diseases [2]. Therefore, an antioxidant intake is needed to reduce the excess of the ROS and thus suppress oxidative damage. An antioxidant is a substance that even at low concentration delays and prevents the oxidation of the substrate [3]. Plants are an important source of natural antioxidants and their compounds have been reported to reduce oxidative stress by acting as an antioxidant [4].

*Carissa edulis* is a plant of the Apocynaceae family which consists of more than 250 genera and 2000 species [5]. It is widely distributed in Africa and in Cameroon; it is found in the Sudano-Sahel savannahs. The plant* Carissa edulis* is reputed in traditional medicine as a good source of medicine for the treatment of rheumatism [6], headaches, epilepsy, gonorrhoea, syphilis, and rabies and is often used as a diuretic [7, 8]. It has also been reported to treat fever, hernia, sickle cell anaemia, ulcer, worm infestation, pain, and inflammation [9, 10]. Several classes of chemical constituents have been isolated, such as sesquiterpenes, cardiac glycosides, phenolic compounds, flavonoids, lignans, sterols, tannins, proanthocyanidins, chlorogenic acid derivatives [5, 11, 12], and 2-hydroxacetophenone [13]. In addition, there are six volatile compounds of* C. edulis* root that have been analysed by GC/MS [14].* C. edulis* has also shown pharmacological activities such as antimicrobial [15], diuretic [7], hypoglycaemic [16], anticonvulsant [17], antiviral [18], anti-inflammatory [19], and antiplasmodial [20, 21].

In this study, the leaf extracts of* C. edulis* were analysed using HPLC; the quantification of total phenolic, flavonoids, and tannins content and the antioxidant capacity as measured by DPPH, ABTS, and FRAP method were evaluated.

## 2. Material and Method

### 2.1. Plant Material

Fresh leaves of* Carissa edulis* were collected in Bogo, in the Diamaré subdivision of the Far Northern Region of Cameroon in the month of June 2016. The botanical identification was done at the National Herbarium of Yaoundé (Cameroon) where voucher specimen was kept under the number 2965/SRFK. The leaves of* C. edulis* collected were washed, dried in the shade, crushed, and then sieved to obtain a fine powder.

### 2.2. Preparation of Extracts

Five hundred grams (500g) of powder were macerated, respectively, with 3 L of ethanol (70% ethanol-30% water), methanol, and water for 2 days. The macerate obtained was filtered and evaporated using a rotary evaporator (BUCHI Rotavapor R-300) at 50°C. After that it was dried in an oven (Heratherm oven). The extracts obtained either hydroethanolic (HEC), methanolic (MEC), or aqueous (AEC) were preserved and used for the determination of compounds and antioxidant activity.

### 2.3. Drugs and Chemicals

Chemicals include Folin-Ciocalteu, sodium carbonate, gallic acid, aluminium chloride, sodium acetate, rutin, vanillin, tannic acid, 2,2-diphenyl-1-picrylhydrazyl (DPPH), ABTS (2,2′-azinobis-(3-éthylbenzothiazoline-6-sulfonique)), potassium ferricyanide, trichloroacetic acid, iron III chloride, ascorbic acid, potassium persulfate, quercetin, acetonitrile (Biochem, Chemopharma), ethanol, methanol, acetone, acetic acid, hydrochloric acid. All chemicals were purchased from Sigma Aldrich St Louis, USA, Daejung, Korea, and local suppliers.

### 2.4. HPLC Analysis

HPLC analysis used for determination of different compounds present in our extracts was performed on a SCHIMADZU LC-20AP system equipped with a binary solvent dispensing pump, an autosampler, and an SPD-20AV UV/visible detector and controlled by the Empower-II software. A reversed-phase RP-U8 OB column (150 mm x 4.6 mm ID, particle size 5 *μ*m) was used for all separations at a column temperature of 30°C. The mobile phase composed of A (1% acetic acid in acetonitrile) and B (1% acetic acid in water) with gradient elution 0 min (5% A), 0-21 min (5-20% A), 21-30 min (20-25% A), 30-32 min (25-100% A), 32-39 min (100-100% A), 39-40 min (100-5% A), and 40-45 min (5-5% A) was used in this study. The injection volume of the sample was 1 *μ*l, and the flow rate was set at 0.6 ml/min. Standards and different extracts were prepared at 1 mg/ml and 5 mg/ml in HPLC grade methanol, respectively. Peaks were determined at 270 nm. Characterization of the peaks was made according to their amplitude. Identification was performed using retention times and available standards. Quantification of the identified compounds was based on the percentage % peak area.

### 2.5. Total Phenolic Content

Total phenolic content was evaluated according to the spectrophotometric method using the Folin-Ciocalteu reagent described by Gao [22] with slight modifications. 0.3 g of different extracts was introduced into a test tube and a volume of 15 ml of 70% ethanol (v/v) was added. The mixture was centrifuged at 3500 rpm for 20 minutes and the supernatants were collected. About 0.02 ml of supernatant was put into a test tube; then 1.38 ml of distilled water and 0.2 ml of Folin-Ciocalteu reagent were added. After standing for 3 minutes, 0.4 ml of sodium carbonate (7.5% Na_2_CO_3_) was added to the mixture. The tubes were vortexed and incubated for 20 minutes in a 40°C water bath and the absorbance was read against a blank at 760 nm. Calibration was performed using a freshly prepared aqueous solution of gallic acid (0.2 g/l). The results were expressed in milligrams equivalent of gallic acid per gram of dry extract.

### 2.6. Total Flavonoid Content

The flavonoids content in the extracts was determined by the method described by Mimica-Dukic [23]. For analysis, 0.25 g of powder of each extract was homogenized with 10 ml of methanol-distilled water-acetic acid extraction solvent (140: 50: 10 v/v). The homogenized mixture was filtered using Whatman No. 1 filter paper. To 0.2 ml of extract solution, 1.8 ml of distilled water and 1 ml of the aluminium chloride reagent (AlCl_3_) (consisting of 133 mg of aluminium chloride crystals and 400 mg of sodium acetate dissolved in 100 ml of the extraction solvent) were added and the whole was vortex mixed and the absorbance was read against a blank at 430 nm. The amount of flavonoids was calculated using a standard solution of rutin (0.1 mg/ml) and the results were expressed in milligrams of rutin per gram of dry extract.

### 2.7. Tannin Content

Tannin was determined using the spectrophotometric method using acidified vanillin [24]. About 1 g of the various extracts was introduced into an Erlenmeyer flask and 15 ml of acetone (10% acetic acid) was added. The mixture was stirred for 15 minutes and then filtered and diluted 20 times with distilled water before the assay. 1 ml of the diluted mixture was added to each test tube coated with aluminium foil to exclude light followed by 3 ml of a freshly prepared solution of 4% vanillin in ethanol (w/v). After stirring, 1 ml of concentrated HCl was added to each tube which was then allowed to stand at room temperature for 15 minutes and the absorbance was read at 500 nm against a blank. The amount of tannins was calculated using a standard solution of tannic acid (5 mg/ml) and the results were expressed in milligrams of tannic acid per gram of solids.

### 2.8. Antioxidant Activity

#### 2.8.1. DPPH Free Radical Scavenging Test

The antioxidant power which is the ability of a given substance to trap a free radical has been determined by the method of Zhang and Hamauzu [25] with some modifications. In a test tube containing 0.5 ml of different concentration (0.1-1 mg/ml) of extract, 2 ml of DPPH (0.1 mM prepared in methanol) was introduced. Then the mixture was stirred for 5 min and incubated in the dark for 60 minutes at room temperature. For the control tube, the methanol was used in place of the extract. The reference used was ascorbic acid at different concentrations. Tests were repeated three times at each concentration level. Absorbance was read at 517 nm. The antioxidant activity was expressed as percent inhibition.

(1) $$\begin{array}{c} I\%=\left[\;\frac{\left(\text{Abs control}-\text{Abs sample}\right)}{\text{Abs control}}\;\right]\times 100 \end{array}$$

IC50 values were determined graphically by the linear regression line.

#### 2.8.2. Inhibition of the Radical ABTS^+^

It is one of the most widely used methods for determining the antioxidant activity of plant extracts; it consists in following the kinetics of discoloration of the ABTS^+^ ion as described by Re et al. [26]. ABTS (2,2′-azinobis-(3-ethylbenzothiazolin-6-sulfonic acid)) was prepared by mixing 0.0384 g of ABTS and 0.00662 g of potassium persulfate (K_2_S_2_O_8_) with 10 ml of distilled water. The mixture was incubated for 16 h at room temperature, protected from light before use. For the actual analysis, the ABTS solution was diluted with ethanol and the absorbance adjusted to 0.700 (± 0.02) at 734 nm and was stable at 30°C (initial OD). In a test tube, 3.0 ml of this diluted ABTS solution and 30 *μ*l of the extract of varying concentration (0.25 mg/ml, 0.1 mg/ml, 0.05 mg/ml, and 0.025 mg/ml) were introduced and agitated to mix. Absorbance reading was taken at 734 nm immediately after agitation. Ascorbic acid was used as an antioxidant reference at the same concentrations as the extracts. The percent inhibition was calculated according to the formula:

(2) $$\begin{array}{c} I\%=\left[\;\frac{\left(\text{Abs control}-\text{Abs sample}\right)}{\text{Abs control}}\;\right]\times 100 \end{array}$$

#### 2.8.3. Ferric Reducing Antioxidant Power (FRAP)

The reducing power of iron (Fe^3+^) in the extracts was determined according to the method described by Oyaizu [27]. 1 ml of the extract was mixed with 2.5 ml of 0.2M phosphate buffer solution (pH 6.6) and 2.5ml of 1% potassium ferricyanide (K_3_Fe(CN)_6_) solution. This was then incubated in a water bath at 50°C for 20 min. Then, 2.5 ml of 10% trichloroacetic acid was added to stop the reaction and the tubes were centrifuged at 3000 rpm for 10 min. To 2.5 ml of supernatant were added 2.5 ml of distilled water and 0.5 ml of an aqueous solution of iron chloride III (0.1% FeCl_3_). The absorbance of the reaction mixture was read at 700 nm against a similarly prepared blank, by replacing the extract with distilled water which made it possible to calibrate the apparatus (UV-VIS spectrophotometer). The positive control was represented by a solution of a standard antioxidant, ascorbic acid whose absorbance was measured under the same conditions as the samples. An increase in absorbance corresponds to an increase in the reducing power of the extracts tested and the results were expressed in grams of ascorbic acid equivalent/100 g of dry extract.

### 2.9. Statistical Analysis

Each set of data was expressed as mean ± ESM (n = 3). The analysis was done by ANOVA followed by Fisher's test and Dunnett's multiple comparison test. The difference between concentrations and extracts was considered significant at p<0.05. The graphical representation of the data and the determination of the IC_50_ were performed using the Graph Pad Prism 5.0 software (Microsoft, USA).

## 3. Results

### 3.1. HPLC Analysis

**Figure 1:**
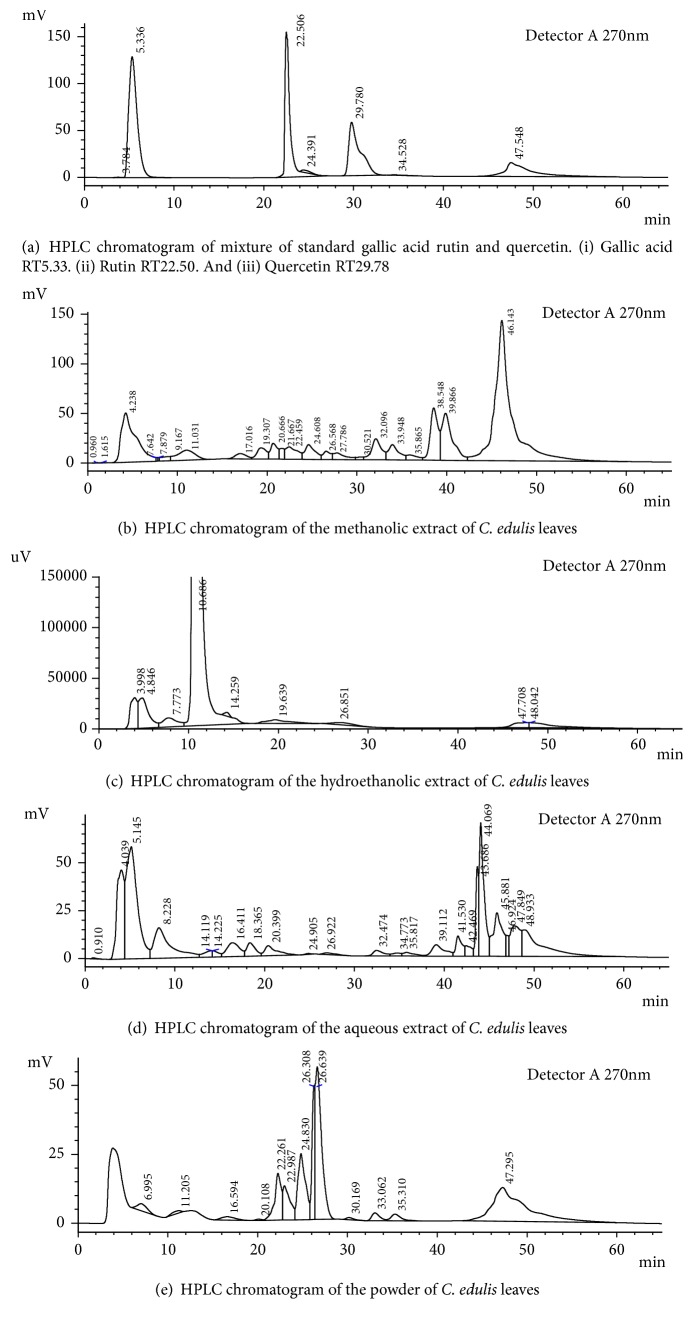


Figure 1(b) shows the HPLC chromatogram of the methanolic extract where 22 peaks are distinguished.  Figure 1(c) shows the chromatogram of the hydroethanolic extract with 9 peaks and Figure 1(d) shows the aqueous extract with 23 peaks. A total of 13 peaks were identified in the chromatogram of the* C. edulis* powder (Figure 1(e)). To find the phenolic compounds present in our extracts, the retention times of each peak of our extracts were compared to that of the standards (Figure 1(a)). The results show that the retention times of gallic acid, rutin, and quercetin are, respectively, RT5.33, RT22.50, and RT29.78 with composition percentages of 37.10%, 27.50%, and 21.64%.

### 3.2. Extraction Yield

The percent yield of extraction is shown in Table 1. The highest percentage was obtained when the leaves were extracted with methanol. The yield of the methanolic extract is significantly higher with 56.83% compared to the hydroethanolic and aqueous extracts which, respectively, have 36.64% and 23.14%.

### 3.3. Total Phenolic Content

**Figure 2:**
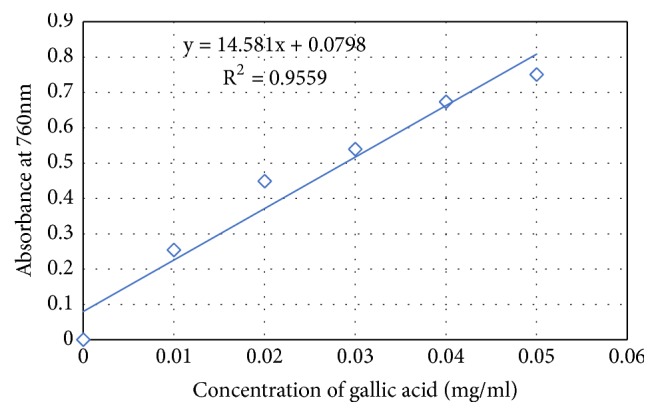
Calibration curve of gallic acid in determination of total phenolic content of* C. edulis*.

The phenolic content of each extract was expressed in milligrams of gallic acid per gram of dry matter and obtained from the calibration curve of gallic acid (Figure 2). The results presented in Table 1 show that the extracts have a significantly high content of polyphenol per gram of dry extract.

### 3.4. Flavonoid Content

**Figure 3:**
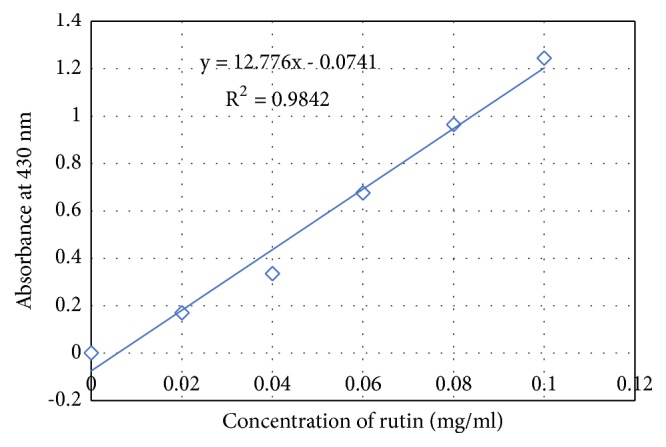
Calibration curve of rutin in determination of flavonoid content of* C. edulis*.

The flavonoid content expressed in mg of rutin per g of dry extract was obtained from the calibration curve of rutin (Figure 3). The hydroethanolic and methanolic extracts have a relatively significant content of 14.84 ± 0.013 mg and 12.02 ± 0.017 mg, respectively, in contrast to aqueous extract and powder that had low content with 5.88 ± 0.120 mg and 5.24 ± 0.017 mg, respectively. The results are shown in Table 1.

### 3.5. Tannins Content

**Figure 4:**
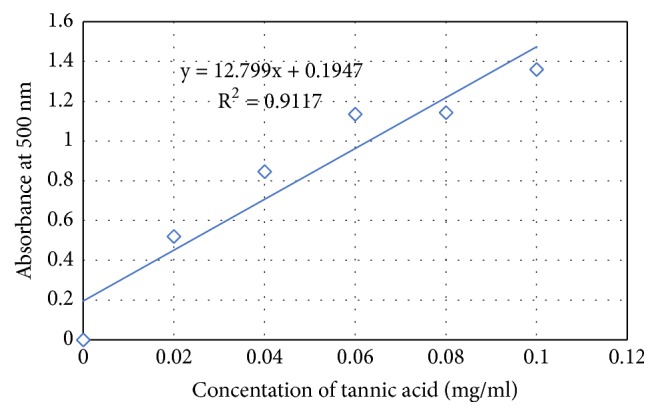
Calibration curve of tannic acid in determination of tannin present in* C. edulis*.

The tannin content of the leaves of* C. edulis* was determined according to the method using acidified vanillin. The results obtained from the standard curve of tannic acid (Figure 4) are expressed per mg of tannic acid per g of dry extract. Table 1 presents the results and it is found that the aqueous extracts have a low tannin content of 16.09 ± 0.043 mg compared to the other hydroethanolic, methanolic, and powder extracts with 26.76 ± 0.048 mg, 34.67 ± 0.042 mg, and 22.41 ± 0.013 mg tannin per gram of plant extract, respectively.

### 3.6. Antioxidant Activity

#### 3.6.1. DPPH Radical Scavenging Activity

**Figure 5:**
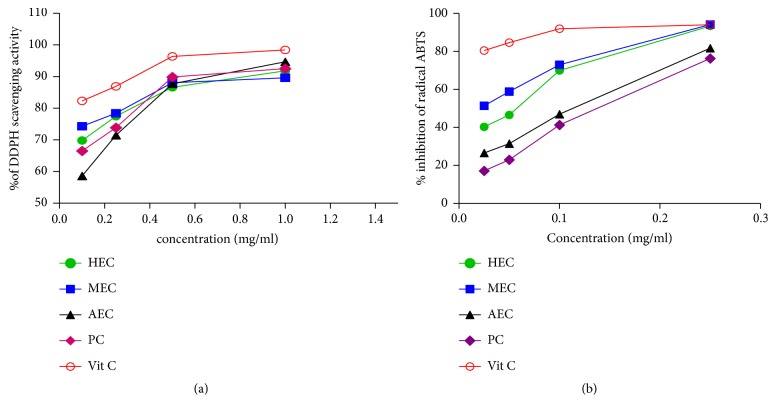
(a) DPPH radical scavenging activity of* C. edulis*. (b) Percentage inhibition of radical ABTS.* HEC*: hydroethanolic extract* Carissa*;* MEC*: methanolic extract* Carissa*;* AEC*: aqueous extract* Carissa*;* PC*: powder* Carissa*;* Vit C*: vitamin C.

The DPPH test determines the ability of a compound or extract to trap a free radical. The results of the DPPH radical scavenging assay show that the various extracts of* C. edulis* possess radical scavenging power (Figure 5(a)) The inhibitory activity of the extracts increases in a dose-dependent manner with the concentration (0.1-1 mg/ml) of 58.63% to 94.67%. All extracts at 0.1 mg/ml to 1 mg/ml showed a significant percentage inhibition compared to vitamin C. The IC_50_ values are shown in Table 2. It defines the effective concentration of the extract that scavenges 50% of the DPPH radical ^−^.

#### 3.6.2. Reduction of Radical ABTS^+^

It is one of the most used tests for determining the antioxidant activity of plant extracts. The results obtained presented in Figure 5(b) show that the percentage of inhibition of the various extracts increases significantly in a dose-dependent manner. Aqueous extract and raw powder of* C. edulis* have relatively lower effect on ABTS inhibition activity (26.25% and 17.16%, respectively) compared to the ascorbic acid which presented 80.41% at the lowest concentration of 0.025 mg/ml. The IC_50_ value of the hydroethanolic extract is significantly comparable to the IC_50_ of the ascorbic acid standard. All data are represented in Table 2.

#### 3.6.3. Ferric Reducing Antioxidant Power

**Figure 6:**
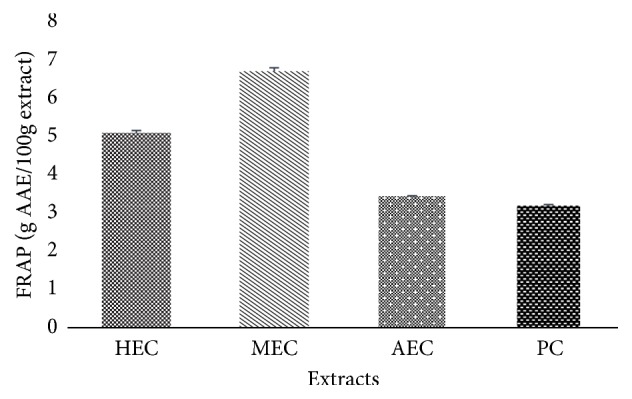
Reducing power of extracts of* C. edulis*.* HEC*: hydroethanolic extract* Carissa*;* MEC*: methanolic extract* Carissa*;* AEC*: aqueous extract* Carissa*;* PC*: powder* Carissa*;* Vit C*: vitamin C. n = 3.

The iron reducing power test based on the reduction of Fe^3+^ iron to Fe^2+^ iron was used in this study to highlight the antioxidant potential of* C. edulis*. The results were calculated from the calibration curve of ascorbic acid and expressed in g of ascorbic acid per 100 g of extracts and presented in Figure 6. This shows that the methanolic extract has a high reducing capacity in the order of 6.73 ± 0.09 g AAE/100g followed by the hydroethanolic extract which is 5.17 ± 0.03 g AAE/100 g.

## 4. Discussion

Plants prove to be an important source of natural antioxidant that fight oxidative stress caused by an increase in free radicals/ROS such as superoxide O^2-^ anion, hydroxyl radical HO^−^, and also peroxide. The medicinal plants used in traditional medicine are particularly interesting for the study of their antioxidant activity. A study has already been done on the antioxidant capacity of the roots of* C. edulis* [19] and fruits [28].

In the present investigation, different extracts of* C. edulis* leaves were evaluated for phenolic compounds and antioxidant capacity. The variations in the antioxidant properties observed may be due to the polarity of the extraction solvents. These results indicated that, because of the diversity of chemical components, their availability for extractable solvents is the basic factor that can influence extraction efficiency [29, 30]. It has also been suggested that the maceration method may be a better choice for the extraction of secondary metabolites.

In most medicinal plants, phenolic compounds such as flavonoids, tannins, and phenols are major contributors to antioxidant activity [31]. These are secondary metabolites found in plants and have a wide range of therapeutic effects. These phenolic antioxidants are potential radical scavengers and the potential to trap free radicals can be explained by hydroxyl phenolic groups [19, 32]. A large amount of total phenolic content, flavonoids, and tannins are found in the leaves of* C. edulis*. A root tea study of* C. edulis* also revealed that this plant contains a high level of phenolic compounds [18, 33]. These results are consistent with the works of Fowsiya and Madhumitha [28], which show that ethanolic and aqueous extracts also have a high content of total polyphenols and flavonoids. These phenolic compounds may be responsible for the antioxidant capacity of* C. edulis*.

The determination of the DPPH antioxidant activity of the extracts and the ascorbic acid standard was evaluated using the spectrophotometer by following the reduction and kinetics of decolorization of the DPPH radical. The percentage of inhibition and the IC_50_ of HEC, MEC, AEC, and PC extracts compared to ascorbic acid show that* C. edulis* has a high antioxidant capacity and that these extracts have the capacity to trap the DPPH radical as the reference antioxidant ascorbic acid. A previous study on the roots showed a percentage inhibition of DPPH of 62.7% [34] and another on fruits a percentage of 80.48% [28]. Antioxidant molecules such as ascorbic acid, tocopherol, flavonoids, and tannins have been shown to induce DPPH decolorization due to their ability to donate hydrogen [35]. This implies that extracts of* C. edulis* can be used for the treatment of pathologies related to oxidative stress.

The antioxidant activity of the extracts was also evaluated by their ability to inhibit the ABTS^*∗*+^ radical obtained from ABTS. This activity increases in a dose-dependent manner with the concentrations of the different extracts. The IC_50_ value of the methanol extract is close to that of the standard. The lower the IC_50_ value, the stronger the antioxidant power.

The total reducing power is another indicator test of the antioxidant activity of natural products. This antioxidant capacity of the extracts could result from the reduction of Fe^3+^ iron to Fe^2+^ based on electron gain which is an important mechanism of action of phenolic antioxidants [1]. The reducing power of the extracts decreases from MEC, HEC, AEC, and PC. The results of Woode [19] show that the hydroethanolic extract of the roots of* C. edulis* has a strong antioxidant power in reducing iron (III) similar to our results as HEC leaves have significant activity. Similarly, the ethanolic and aqueous extracts of the fruits showed a good reducing power [28]. In the FRAP test, the fact that Fe^3+^ accept an electron to become Fe^2+^ is responsible for the resulting green colour. The higher intensity of the colour shows that the extract has a significant reducing power. It is suggested that there is a direct correlation between antioxidant activity and reducing power of the components of some plants [36]. It is noted in this work that the results obtained show that the methanolic and hydroethanolic extracts of* C. edulis* have this ability to donate an electron to become more stable and thus act as antioxidant substances. Suitable solvents often used for the extraction of phenolic compounds from plant materials include alcohols (methanol and ethanol), acetone, and ethyl acetate [37].

HPLC can be used to separate, quantify, and identify polyphenols [38]. The HPLC chromatograms of the extracts have shown various peaks of compounds. All these compounds contained in the extracts of* C. edulis* may account for its antioxidant capacity. The most important compounds identified in our study are gallic acid, quercetin, and rutin. These were obtained by comparing their retention time with those of standard and the spectral characteristic of the peaks. The gallic acid was higher in the aqueous extract than the others with a percentage of composition of 22.63%. Quercetin was found more in raw powder and methanolic extract. Gallic acid, quercetin, and rutin are phenolic compounds; this can justify the antioxidant activity of* C. edulis*.

## 5. Conclusion

The determination of the antioxidant activity and the quantification of the bioactive compounds of extracts obtained from* Carissa edulis* were evaluated. This work revealed to us that the different extracts of* Carissa edulis*, namely, the hydroethanolic, methanolic, and aqueous extracts, and the powder contain a high content of polyphenols, flavonoids, and tannins. Phenolic compounds such as gallic acid, quercetin, and rutin were identified and may be related to the antioxidant capacity of these plant extracts as measured by radical scavenging activity (DPPH and ABTS) ferric reducing antioxidant power.

## Figures and Tables

**Table 1:** Percent yield, content of total polyphenols, flavonoids, and tannins contained in *C. edulis*.

Extracts	% Yield	Total phenolic content (mg GAE/g extract)	Total flavonoid (mg Rutin/g extract)	Tannin (mg TAE/g extract)
Hydroethanolic	36.64	139.27 ± 0.014	14.84 ± 0.013	26.76 ± 0.048
Methanolic	56.83	146.82 ± 0.001	12.02 ± 0.017	34.67 ± 0.042
Aqueous	23.14	147.05 ± 0.014	5.88 ± 0.120	16.09 ± 0.043
Powder		144.42 ± 0.009	5.24 ± 0.017	22.41 ± 0.013

**Table 2:** IC_50_ values of *C. edulis* extracts and vitamin C in DPPH scavenging, ABTS activity, and reducing power assays.

	IC_50_ values	
Extracts	DPPH scavenging	Radical ABTS^+^
HEC	0.321±0.019	0.09±0.003
MEC	0.310±0.027	0.123±0.005
AEC	0.304±0.013	0.153±0.007
PC	0.307±0.013	0.135±0.006
Vit C	0.313±0.005	0.061±0.001

## Data Availability

All results presented in this study were carried out by authors and data used as references were properly cited.
